# ID 159 - Qualidade de Relato de Estudos de Custo-Minimização: uma metarrevisão sistemática

**DOI:** 10.5327/2237-9622.2025.v34s1.159

**Published:** 2025-11-25

**Authors:** Aline Rocha, Denis Satoshi Komoda, Ana Carolina Pereira Nunes Pinto, Nayara Castelano Brito, Aíla Coelho do Carmo, Alexia Gabriela Vieira, Lays Marra, Wendel Mombaque

**Keywords:** qualidade metodológica, qualidade do relato, análise de custo-minimização, revisão sistemática

## Abstract

**Introdução:**

Análises de custo-minimização são ferramentas convenientes para mostrar a comparação de valor monetária entre tecnologias em saúde equivalentes. No entanto, o embasamento teórico para a presunção de equivalência pode ser profusamente em diferentes estudos. Além disso, a heterogeneidade nos padrões de relato de diferentes estudos pode também comprometer a transparência e afetar a confiança nos relatos desses estudos. Com o objetivo de avaliar a qualidade de relato e explorar as características metodológicas quanto ao embasamento da presunção de equivalência em estudos de custo-minimização, esta pesquisa será baseada em um método de metarrevisão sistemática.

**Método:**

Uma busca estruturada adaptada para cada base de dados será conduzida em sete bases de literatura acadêmica especializada, utilizando termos relacionados à "análise de custo-minimização". A seleção dos estudos será realizada em duas fases (por leitura de título e resumo, e por leitura de texto completa), por dois pesquisadores independentes em cada fase. Os dados serão extraídos em planilhas predefinidas e conterão minimamente informações sobre as características da publicação (e.g. autor, ano, país de condução do estudo e idioma), características metodológicas (e.g. embasamento da presunção de equivalência, tecnologias comparadas), características do relato (e.g. aplicação de diretrizes de qualidade de relato, conflito de interesse e fonte de financiamento do estudo).

**Resultados:**

Os dados serão analisados por meio de frequências absolutas e relativas. Complementarmente, uma análise por regressão logística avaliará as seguintes variáveis independentes: preocupações quanto à qualidade do relato (sim ou não), preocupações quanto ao método empregado (sim ou não) e financiamento pela indústria farmacêutica (sim ou não). Os resultados serão reportados seguindo as orientações do "PRISMA Statement 2020", e serão apresentados de forma narrativa, gráfica e tabular. A difusão de conhecimento será conduzida por meio de publicação em revista acadêmica, submissão de trabalhos em congressos, participação em webinários ou seminários. Os participantes do presente projeto declaram não ter conflito de interesse com o projeto.

**Conclusão:**

A análise de custo-minimização é amplamente utilizada para comparar tecnologias de saúde que são consideradas equivalentes em termos de eficácia. No entanto, a suposição de equivalência frequentemente carece de fundamentação teórica consistente, o que pode comprometer a validade das conclusões. A variabilidade nos padrões de relato entre estudos adiciona outra camada de complexidade, dificultando a comparação direta e a interpretação dos resultados. Este estudo, ao empregar uma metarrevisão sistemática, buscará não apenas avaliar a qualidade do relato, mas também investigar as bases metodológicas que sustentam a presunção de equivalência. A inclusão de variáveis como preocupações com a qualidade do relato e financiamento pela indústria farmacêutica na análise por regressão logística poderá revelar influências importantes sobre os resultados reportados.

